# Assessing the Mediating Role of Internal Migration in Bushfire‐Related Changes in the Distribution of Australia's Medical Practitioners

**DOI:** 10.1002/puh2.70436

**Published:** 2026-11-03

**Authors:** Xin Lin, Ross Bailie, Peter Radchenko, Geoffrey Morgan, Anirban Chakraborti, K. Shuvo Bakar

**Affiliations:** ^1^ School of Public Health The University of Sydney Camperdown Australia; ^2^ Sydney Business School The University of Sydney Darlington Australia; ^3^ School of Computational & Integrative Sciences Jawaharlal Nehru University New Delhi India; ^4^ Institute for Interdisciplinary Research New Delhi India

**Keywords:** bushfire exposure, generalised estimating equation (GEE), health workforce, internal migration, mediation analysis

## Abstract

**Background:**

Climate‐driven bushfires increasingly shape community health needs and challenge medical workforce capacity in Australia. Although population mobility can influence practitioner availability, it is unclear whether bushfires indirectly affect medical practitioner workforce distribution by prompting internal migration. Understanding these dynamics is critical for informing climate‐resilient health‐workforce policies.

**Methods:**

We analysed a longitudinal panel of regional and remote Australian Local Government Areas (LGAs) from 2013 to 2021 using generalised estimating equations (GEEs) causal mediation analysis. Bushfire exposure was assessed using three complementary measures: (i) binary (any occurrence), (ii) cumulative (repeated events) and (iii) severity‐based (intensity of exposure). Outcomes were full‐time equivalent (FTE) medical practitioner, adjusted for remoteness and socio‐economic conditions. Internal migration was included as a potential mediator.

**Results:**

Across all exposure metrics, bushfire‐affected LGAs exhibited significantly higher medical practitioner FTE rates. Cumulative exposure showed a positive association (0.29, *p* = 0.02), with severity‐weighted exposure yielding a similar directional effect (1.32, *p* = 0.04). Although internal migration was positively associated with FTE rates, it did not significantly mediate the relationship between bushfire exposure and workforce supply.

**Conclusions:**

Bushfire exposure is associated with higher medical practitioner FTE rates in affected LGAs. However, internal migration does not appear to meaningfully mediate this relationship for our study region. For extreme and consecutive bushfires, defined as bushfire occurrence in at least five of the past 10 years, the evidence for mediation through population movement is limited. These findings suggest that changes in the medical workforce following bushfires are more likely driven by direct health system policies rather than by broader internal migration patterns. Planning for future climate‐related disasters should consider mechanisms that support responsive allocation and retention of medical practitioners, particularly in regional and remote areas with historically lower workforce levels.

## Graphical abstract

Bushfire exposure was associated with higher medical practitioner workforce supply across Australian LGAs (2013–2021). Although internal migration was positively associated with workforce levels, it did not meaningfully mediate this relationship. Findings highlight the need for responsive workforce allocation and retention strategies, particularly in regional and remote areas affected by climate‐related disasters.

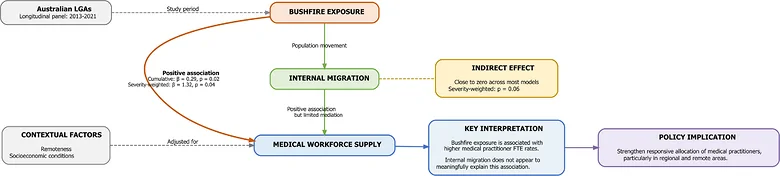

## 1 Introduction

Migration is rarely driven by a single force; rather, it emerges from the interaction of economic opportunity, social conditions and environmental change [1]. Although employment, income and social networks continue to anchor most mobility decisions, environmental shocks are increasingly recognised as catalysts that disrupt established settlement patterns [2, 3]. Across diverse contexts, disasters, including earthquakes, floods and droughts, have triggered large‐scale population movements, often abruptly and with limited capacity for communities to adapt in place [4]. In recent decades, the scale of such displacement has highlighted a broader shift: Environmental risk is no longer peripheral to migration systems but has become an integral and growing influence.

Climate change is intensifying these dynamics by altering the frequency, severity and geographic reach of extreme events, reshaping both where people can live and how communities function [5, 6]. In Australia, rising temperatures and increasingly extreme fire weather conditions have intensified the risk and impact of bushfires [7, 8], exposing populations to repeated disruption. Climate‐related events, including bushfires, floods and heatwaves, have already demonstrated substantial impacts on communities and health systems [9, 10, 11]. The 2019–2020 ‘Black Summer’ fires, for example, displaced tens of thousands of residents, damaged critical infrastructure and disrupted access to healthcare services [12]. These disruptions are not only immediate; they may also initiate longer term adjustments in settlement patterns as households reassess risk, livelihoods and access to essential services.

Such mobility responses have important implications beyond population redistribution. Internal migration, whether temporary or permanent, can reconfigure the demand for and supply of critical services, including healthcare. Population inflows may increase pressure on already stretched urban systems, whereas outflows from rural or fire‐affected areas can undermine the viability of local services. These shifts are particularly consequential in Australia, where longstanding disparities in healthcare access persist between cities and rural regions [13]. Even modest changes in population distribution can amplify existing inequities, especially where workforce supply is already fragile.

At the same time, the health workforce is itself dynamic. Medical practitioners respond to many of the same forces shaping broader migration patterns, including professional opportunities, working conditions and community viability. Migration patterns significantly influence the distribution of health professionals, and workforce mobility can exacerbate geographic imbalances [14]. Evidence from Australia and comparable settings suggests practitioners tend to concentrate in urban and better resourced areas, contributing to persistent shortages in rural and remote communities [13, 15]. When environmental shocks disrupt local economies, infrastructure and living conditions, practitioners’ location decisions may be further influenced, either directly or indirectly through changes in population demand and service sustainability.

Disasters add another layer of complexity [16], directly affecting health service capacity and practitioner availability. Bushfire smoke has been linked to increased hospital admissions, particularly for respiratory conditions [17], and surges in clinical demand during acute fire events [18]. Infrastructure damage can disrupt service delivery, and in some Aboriginal community‐controlled health services, facilities have been repurposed as evacuation or supply hubs rather than operating as routine care providers [19]. Additionally, economic losses in small rural communities affected by bushfires or drought can undermine the sustainability of local health services, intensifying pre‐existing workforce inequities [20].

Bushfires, therefore, sit at the intersection of these processes. They can simultaneously prompt population movement, alter healthcare demand and disrupt the conditions that sustain local health services. This raises a critical but underexplored question: To what extent do bushfires shape the geographic distribution of the medical practitioner workforce not only through direct impacts but also indirectly through the migration patterns they induce?

This study addresses this gap by examining a causal pathway linking bushfire exposure, internal migration and the distribution of medical practitioner full‐time equivalent (FTE) rates across Local Government Areas (LGAs) in Australia. Migration, environmental shocks and healthcare workforce distribution can be understood as co‐evolving, interdependent processes characterised by feedback loops, nonlinearity and emergent outcomes of a complex adaptive system [21]. Rather than operating in isolation, these components form a complex socio‐environmental system in which small perturbations, such as localised bushfire events, can propagate through migration dynamics to produce disproportionate and spatially uneven impacts on health service capacity. Specifically, it investigates whether internal migration mediates the relationship between bushfire events and workforce distribution. By situating environmental shocks within a broader system of population mobility and service provision, this research provides an integrated understanding of how climate‐related disasters can reshape health system capacity. Knowing whether bushfires indirectly change where medical practitioners work through population movement is important for creating policies that address both immediate challenges and longer term shifts in communities and healthcare needs.

## 2 Methods

### 2.1 Research Questions and Hypotheses

This study investigates a critical policy challenge: how climate‐driven bushfire events influence the availability and distribution of medical practitioners across Australian LGAs and whether internal migration functions as a pathway mediating these effects. Understanding these dynamics is essential for designing resilient health‐workforce strategies, particularly as bushfires are projected to become more frequent and severe under climate change scenarios [7, 8].

To capture the multiple ways in which bushfires may affect communities and health systems, the analysis examines three complementary specifications of bushfire occurrence:

- *Binary exposure model*: This specification differentiates LGAs that experienced any bushfire activity from those without exposure. Although most bushfire events last only a few days or a week, we assume this metric is more closely associated with acute workforce responses, such as rapid adjustments in staffing, temporary redeployments or short‐term service coverage. Although the direct link between a single bushfire event and immediate workforce decisions cannot be fully observed in the data, prior research suggests that sudden disasters can prompt short‐term redistribution of health resources [17, 18].
- *Cumulative exposure model*: This specification captures repeated or long‐term exposure to bushfires over the study period. We assume this metric reflects longer term workforce responses, such as sustained shifts in practitioner allocation, recruitment and retention in affected regions. Evidence from both Australia and other contexts indicates that recurrent disasters can influence longer term migration and staffing patterns, potentially altering workforce stability in chronically affected communities [13, 20].
- *Severity exposure model*: This measure identifies LGAs as severely affected if bushfires occurred in over half of the past 10 years, reflecting sustained high‐intensity exposure. We assume this metric is more closely related to workforce responses in severely exposed communities, which may experience acute service disruption, infrastructure damage and higher population displacement. High‐risk communities, defined by either chronic exposure or severe bushfire events, are likely to face distinct challenges in maintaining consistent medical service provision [12, 19].

Although the assumptions linking each bushfire metric to workforce responses are supported in empirical evidence of disaster‐driven mobility and health‐service disruption, this study contributes novel insight by formally testing whether internal migration mediates these effects. Understanding these pathways is critical for strengthening climate‐ready workforce planning and ensuring that resources are allocated to communities most likely to experience both immediate and sustained workforce pressures in Australia.

### 2.2 Data

This study conducted a secondary analysis of a longitudinal panel dataset containing 343 regional (inner and outer) and remote (includes very remote) Australian LGAs from 2013 to 2021.

*Internal Migration:* Annual net internal migration at the LGA level was obtained from the Australian Bureau of Statistics (ABS) Data Explorer [22], capturing the number of residents moving into and out of each LGA within Australia. These data provide a consistent measure of population mobility over time and across regions, enabling analysis of migration as a potential mediator between environmental shocks and workforce distribution.

*Bushfire Occurrence:* Bushfire data were sourced from the Australian Government Disaster Assist database [23], which records officially declared natural disasters by year and region. The number of declared bushfire events per LGA was extracted for each year of the study period. From these records, three exposure metrics were constructed: (i) a binary indicator, (ii) cumulative exposure reflecting repeated or long‐term exposure and (iii) severity‐based exposure capturing the intensity of events. These measures align with prior research on disaster‐driven population and workforce dynamics [18, 20].

*Socio‐Economic and Geographic Context:* Socio‐economic conditions were measured using the ABS Socio‐Economic Indexes for Areas (SEIFA) [22], including the Index of Relative Socio‐economic Advantage and Disadvantage (IRSAD) and the Index of Economic Resources (IER). Remoteness area (RA) was classified according to the Australian Statistical Geography Standard (ASGS), allowing differentiation between urban, regional and remote LGAs. These covariates account for structural factors that influence both migration and health‐workforce distribution [13].

*Medical Workforce:* The primary outcome variable was the number of FTE medical practitioners per 100,000 population in each LGA. Data on the medical workforce were obtained from the Health‐Workforce Data Tool [24], which provides annual counts of FTE medical practitioners registered with the Australian Health Practitioner Regulation Agency (AHPRA) and actively practising within each LGA from 2013 to 2021. FTE metrics enable a standardised assessment of workforce capacity that accounts for variations in employment status and part‐time practice. The FTE measure reflects the usual geographic distribution of the Australian medical workforce and is intended to represent the established workforce available within each location. It does not capture short‐term or temporary workforce deployments made under emergency response arrangements, including deployments coordinated through emergency agencies in response to disasters, emergencies or other surge events.

### 2.3 Statistical and Mediation Analysis

Descriptive statistics were used to summarise all key variables. Continuous variables are presented as means with standard deviations (SD), whereas categorical variables are reported as counts and percentages. Pearson correlation coefficients and two‐sample *t*‐tests were used to explore preliminary associations between bushfire exposure, internal migration and medical practitioner FTE rates.

To evaluate the relationships between bushfire exposure and medical practitioner workforce density, three analytical models were fitted to estimate total, direct and indirect associations across Australian LGAs from 2013 to 2021. Bushfire occurrence was evaluated using three measures: (i) a binary indicator, (ii) a cumulative count of declared bushfire events and (iii) a severity measure. Covariates were screened through preliminary regression models to assess multicollinearity and determine their contribution to model fit. Due to the longitudinal repeated observations, generalised estimating equations (GEEs) based analysis was used [25]. The final adjusted models included RA, IRSAD and IER, which exhibited acceptable multicollinearity (variance inflation factor <5). Associations between bushfire exposure, internal migration and medical workforce density were further evaluated using GEE‐based mediation analysis [26, 27]. Although this approach estimates the average causal mediation effect (ACME), average direct effect (ADE) and total effect, it is important to note that, in this observational study, these estimates represent associational pathways rather than definitive causal effects. Specifically, ACME captures the association between bushfire exposure and medical practitioner FTE rates that is statistically linked to internal migration, whereas ADE reflects the association not explained by migration, and the total effect is the sum of these components. Nonparametric bootstrap resampling (1000 simulations) was used to generate 95% confidence intervals and *p* values. Robust standard errors accounted for heteroscedasticity and repeated observations within each LGA. Model diagnostics, including inspection of residuals and variance inflation factors (VIF < 5), indicated acceptable model fit and low multicollinearity.

Figure 1 illustrates the proposed mediation framework, where, bushfire exposure (*X*) is associated with changes in medical practitioner supply (*Y*) both directly and indirectly through internal migration (*M*). Remoteness and socio‐economic indices (IRSAD and IER) are included as potential confounders that may influence the exposure, the mediator and the outcome. Although the analysis quantifies these pathways, all findings should be interpreted as associations rather than causal effects, given the observational nature of the data and the limitations inherent to non‐experimental designs.

**FIGURE 1:**
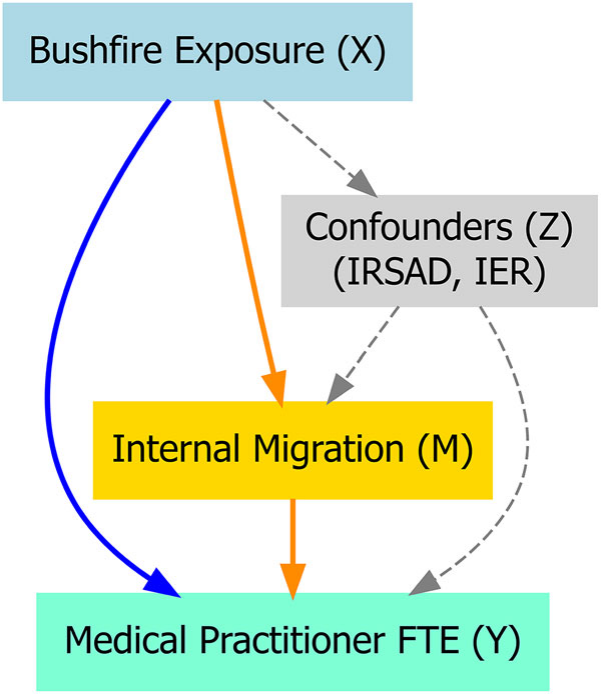
Pathways for causal mediation analysis. FTE, full‐time equivalent; IER, Index of Economic Resources; IRSAD, Index of Relative Socio‐economic Advantage and Disadvantage.

The mediation models were estimated across time (t=2013,…,2021), with observations clustered within LGAs (i=1,…,n), using GEEs to account for within‐LGA correlation. The models were specified as follows:

$$\mathrm{FT}E_{\mathrm{it}}=c\cdot \mathrm{Bushfire}\;\mathrm{Exposur}e_{\mathrm{it}}+Z_{\mathrm{it}}\gamma +\theta$$

This model estimates the total effect of bushfire exposure on medical practitioner FTE rate.

$$\mathrm{Internal}\;\mathrm{Migratio}n_{\mathrm{it}}=a\cdot \mathrm{Bushfire}\;\mathrm{Exposur}e_{\mathrm{it}}+Z_{\mathrm{it}}\gamma^{\prime }+\theta^{\prime }$$

This model estimates the association between bushfire exposure and internal migration.

$$\mathrm{FT}E_{\mathrm{it}}=c^{\prime }\cdot \mathrm{Bushfire}\;\mathrm{Exposur}e_{\mathrm{it}}+b\cdot \mathrm{Internal}\;\mathrm{Migratio}n_{\mathrm{it}}+Z_{\mathrm{it}}\gamma^{\prime \prime }+\theta^{\prime \prime }$$

This model estimates the direct effect of bushfire exposure on medical practitioner FTE distribution and the indirect effect mediated through internal migration. In these models, a represents the effect of bushfire exposure on internal migration, b is the effect of migration on practitioner supply, c is the total effect of bushfire exposure on FTE, $c^{\prime }$ is the direct effect after accounting for migration, Z represents covariates (RA, IRSAD, IER), and θ is the intercept. The ACME is calculated as a×b, the ADE is $c^{\prime }$, and the total effect is the sum of both: $c=a\times b+c^{\prime }$. All analyses were performed in R (version 4.5.3) programming language.

## 3 Results

### 3.1 Descriptive Statistics

The dataset included 3087 observations. Overall, 11.4% of LGAs experienced at least one bushfire event (see Table 1). However, when looking at total exposure over time, only a small number of LGAs were affected multiple times. Most LGAs (75%) were in regional areas, which often have fewer healthcare workers and known workforce shortages. There was a wide range in both workforce levels and population movement across LGAs. Medical practitioner FTE rates ranged from 0 to 2061 per 100,000 people, showing large differences in workforce availability. Internal migration also varied greatly, on average from a loss of 2848 people to a gain of 6392 people per LGA per year. This shows that both healthcare access and population movement differ a lot between areas. We found that LGAs with bushfires had higher average FTE rates than those without bushfires (*p* = 0.002). This suggests a positive relationship between bushfires and medical practitioner workforce levels. However, the binary bushfire exposure was not related to internal migration (*p* = 0.97), meaning bushfires did not appear to affect population movement.

**TABLE 1:** Descriptive statistics of study variables.

Variable	Type/Category	Mean (SD)^a^	*N* (%)^a^
Medical practitioner (FTE)	Continuous	214.93 (201.6)	—
Bushfire occurrence	No	—	2736 (88.6)
	Yes	—	351 (11.4)
Bushfire cumulative occurrence	Continuous	0.84 (1.4)	—
Bushfire occurrence severity	Less severe (<5)	—	2999 (97.1)
	Severe (≥5)	—	88 (2.9)
Internal migration (per 100 persons)	Continuous	1.38 (7.1)	—
Remoteness area (RA)	Regional	—	2313 (75.0)
	Remote	—	774 (25.0)
IRSAD	Continuous	4.93 (2.5)	—
IER	Continuous	5.40 (2.7)	—

Abbreviations: FTE, full‐time equivalent; IER, Index of Economic Resources; IRSAD, Index of Relative Socio‐economic Advantage and Disadvantage; SD, standard deviations.

^a^ If the variable is categorical, then we used ‘*N* (%)’ and if the variable is continuous, then we used ‘Mean (SD)’.

### 3.2 Model‐Based Results

#### 3.2.1 Binary Exposure

Results from the adjusted GEE models (see Table 2) showed that LGAs affected by bushfires had significantly higher medical practitioner supply, with an estimated increase of 0.74 (*p* = 0.01). Bushfire exposure was negatively associated with internal migration, but this relationship was not statistically significant (−0.64, *p* = 0.17), suggesting no clear evidence that bushfires influenced population movement. In the full model that included both bushfire exposure and migration, the positive association between bushfire exposure and FTE rates remained (0.78, *p* < 0.01). Unlike the results we obtained from bivariate analyses, in the model‐based analyses, internal migration was positively associated with practitioner supply (0.06, *p* < 0.01). As illustrated in Figure 2a, the total effect of bushfire exposure on workforce availability was clearly positive. Mediation analysis indicated that the ACME through internal migration was minimal and not statistically significant (−0.04, *p* = 0.094), whereas the ADE remained significant. These findings indicate that bushfire exposure is linked to higher FTE medical practitioner workforce supply. However, the indirect effect through internal migration was very small and not statistically significant, whereas the direct effect remained significant.

**TABLE 2:** Causal mediation generalised estimating equation (GEE) model estimates for binary, cumulative and severe bushfire exposures, considering internal migration as the mediating pathway.

	FTE rate (direct)	Migration (mediator)	FTE rate (overall)
	GEE‐estimate (standard error)[*p* value]		
**Binary exposure**			
Bushfire			
No = 0			
Yes = 1	0.74 (0.29)[0.01]	−0.64 (0.47)[0.17]	0.78 (0.29)[<0.01]
Internal migration	—	—	0.06 (0.03)[<0.01]
Remoteness area			
Regional = 0			
Remote = 1	−3.71 (0.83)[<0.01]	−1.40 (0.49)[<0.01]	−3.63 (0.84)[<0.01]
IRSAD	1.79 (0.32)[<0.01]	−0.22 (0.22)[0.03]	1.81 (0.32)[<0.01]
IER	−1.92 (0.30)[<0.01]	0.70 (0.20)[<0.01]	−1.96 (0.29)[<0.01]
			
ADE		0.80 (0.21)[<0.01]	
ACME		−0.03 (0.02)[0.10]	
Total effect		0.77 (0.20)[<0.01]	
**Cumulative Exposure**			
Bushfire(cumulative)	0.29 (0.13)[<0.01]	−0.64 (0.50)[0.95]	0.29 (0.13)[0.02]
Internal migration	—	—	0.06 (0.02)[0.03]
Remoteness area			
Regional = 0			
Remote = 1	−3.54 (0.85)[<0.01]	−1.34 (0.58)[<0.01]	−3.47 (0.86)[<0.01]
IRSAD	1.80 (0.32)[<0.01]	−0.22 (0.22)[0.31]	1.81 (0.32)[<0.01]
IER	−1.93 (0.30)[<0.01]	0.70 (0.19)[<0.01]	−1.97 (0.29)[<0.01]
			
ADE		0.29 (0.05)[<0.01]	
ACME		−0.001 (0.007)[0.84]	
Total effect		0.29 (0.05)[<0.01]	
**Severity Exposure**			
Bushfire			
Less = 0			
Severe = 1	1.38 (0.68)[0.04]	1.13 (1.37)[0.41]	1.32 (0.65)[0.04]
Internal migration	—	—	0.06 (0.03)[0.03]
Remoteness area			
Regional = 0			
Remote = 1	−3.74 (0.83)[<0.01]	−1.28 (4.8)[<0.01]	−3.67 (0.84)[<0.01]
IRSAD	1.80 (0.32)[<0.01]	−0.21 (0.22)[0.33]	1.81 (0.32)[<0.01]
IER	−1.92 (0.30)[<0.01]	0.69 (0.19)[<0.01]	−1.96 (0.29)[<0.01]
			
ADE		1.31 (0.33)[<0.01]	
ACME		0.07 (0.03)[0.06]	
Total effect		1.37 (0.34)[<0.01]	

Abbreviations: ACME, average causal mediation effect; ADE, average direct effect; FTE, full‐time equivalent; IER, Index of Economic Resources; IRSAD, Index of Relative Socio‐economic Advantage and Disadvantage.

**FIGURE 2:**
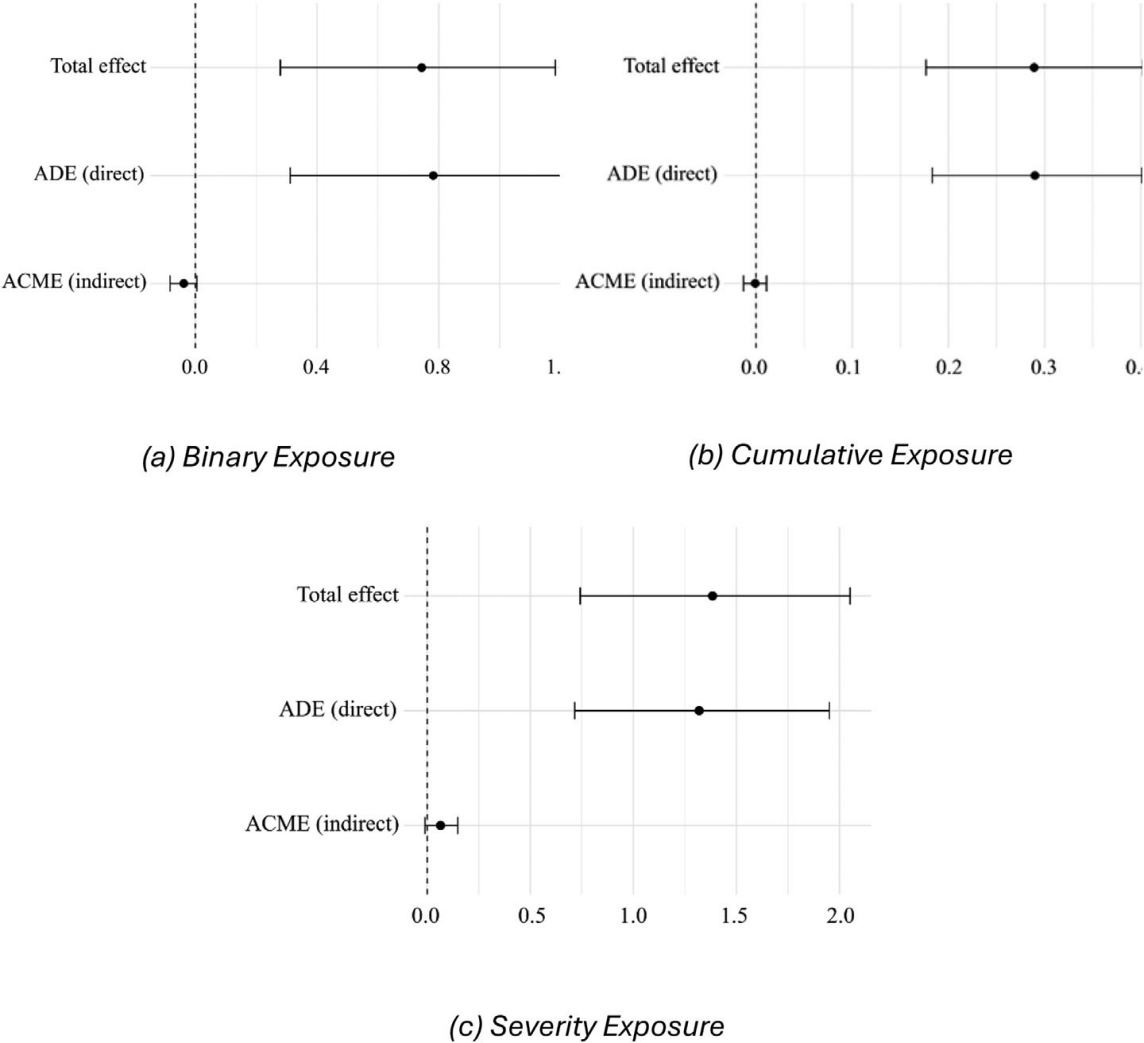
Casual mediation analysis showing total effect, average direct effect (ADE) and average causal mediation effect (ACME) for (a) binary, (b) cumulative and (c) severity bushfire exposures on medical practitioner full‐time equivalent (FTE) rates.

#### 3.2.2 Cumulative Exposure

When cumulative bushfire exposure was considered, a consistent positive association with FTE medical practitioner supply was observed (see Table 2). Each additional bushfire event was associated with an increase of 0.29 in FTE rates (*p* = 0.02). There was no meaningful association between cumulative bushfire exposure and internal migration (−0.64, *p* = 0.95). In the full model, both cumulative exposure (0.29, *p* = 0.02) and internal migration (0.06, *p* = 0.03) were positively associated with practitioner supply. This suggests that LGAs experiencing more frequent bushfires and higher net migration tended to have greater workforce availability. However, the mediation analysis showed that the indirect effect through migration was small and not statistically significant, whereas the direct effect remained significant (also see Figure 2b). This indicates that repeated bushfire exposure influences workforce supply primarily through direct pathways rather than through migration.

#### 3.2.3 Severity Exposure

Using the severity measure, LGAs classified as experiencing severe bushfires had higher medical practitioner supply, with an estimated increase of 1.38 (*p* = 0.04), see Table 2. In the full model, the association between severe bushfire exposure and workforce supply remained positive (1.32, *p* = 0.04). Internal migration again showed a small but significant positive association with FTE rates (0.06, *p* = 0.03). Interestingly, we observe that compared with the other bushfire exposure measures (i.e., binary and cumulative), the ACME of bushfire severity exposure through internal migration provided some evidence of a potential indirect effect (*p* = 0.06), whereas the ADE remained positive and significant (*p* < 0.001; see Figure 2c). These findings suggest that severe bushfire exposure was associated with higher practitioner supply, primarily through direct pathways. Here, the marginal indirect effect mediated through internal migration should be interpreted considering its limited consistency across the other measures of bushfire exposure. Given this potential indirect evidence for mediation, we further examine the robustness of this indirect effect to potential unmeasured mediator‐outcome confounding in the sensitivity analysis presented in the following section.

### 3.3 Sensitivity Analysis

To further assess the robustness of the mediation findings, sensitivity analyses were conducted to examine how potential unmeasured mediator‐outcome confounding might influence the estimated ACMEs. This analysis provides an indication of how strong such unmeasured confounding would need to be to alter the observed mediation effects. Figure 3 presents the sensitivity analyses across the three bushfire exposure models. The top row illustrates the results across different values of ρ, representing the assumed residual correlation between the mediator and outcome models, whereas the bottom row presents the corresponding $R^{2}$ measures, which characterise the strength of hypothetical unmeasured confounding in terms of variance explained.

**FIGURE 3:**
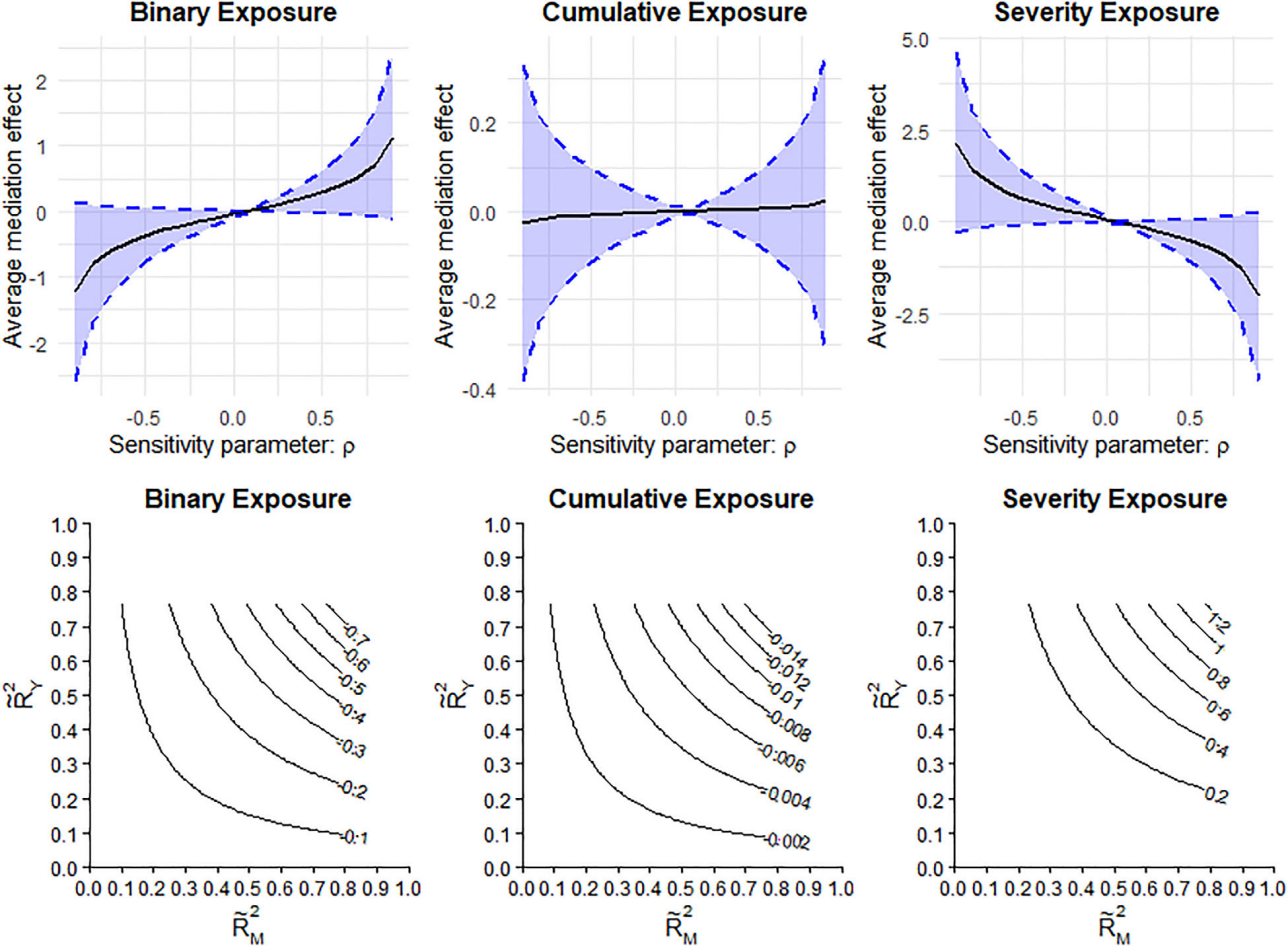
Sensitivity analysis for average causal mediation effect (ACME) for binary (Column 1), cumulative (Column 2) and severity (Column 3) bushfire exposures on medical practitioner full‐time equivalent (FTE) rates.

We see that, under the assumption of no unmeasured mediator‐outcome confounding (ρ=0), the estimated ACME was −0.03 (95% CI: −0.08 to 0.01) for binary bushfire exposure, −0.001 (95% CI: −0.012 to 0.010) for cumulative exposure and 0.07 (95% CI: −0.01 to 0.14) for severity exposure. Across all three exposure measures, the point estimate of the ACME approached and crossed zero at approximately ρ=0.10, corresponding to $\rho^{2}\approx 0.01$. Thus, the estimated mediation effects were generally small and were sensitive to relatively modest departures from the assumption of no unmeasured mediator‐outcome confounding. In particular, the sensitivity plots indicate that a modest degree of residual association between unmeasured mediator‐outcome confounding and the mediator and outcome could attenuate the estimated ACME to zero. The contour plots further show that increasingly large departures from the no‐confounding assumption would be required to produce larger changes in the estimated mediation effects. These findings suggest that the evidence for mediation should be interpreted cautiously, given the relatively small magnitude of the estimated ACMEs and their sensitivity to modest levels of potential unmeasured mediator‐outcome confounding.

## 4 Discussion

This study examined the relationships between bushfire exposure, internal migration and medical practitioner workforce supply (FTE rate) across LGAs using a GEE causal mediation framework. Across all exposure measures, binary, cumulative and severity, bushfire effect was consistently associated with increased medical practitioner FTE rates. Mediation analyses showed that these effects were driven predominantly by ADE, with little evidence of meaningful ACME through internal migration. However, we find that for severity bushfire exposure, the indirect effect is marginally significant. The results reflect significant LGA variability in health‐workforce distribution and population movement.

The positive associations between bushfire exposure and workforce supply may be partly explained by responses to disaster‑driven increases in health service demand. Major fire seasons in Australia, particularly the 2019–20 ‘Black Summer’, had substantial short‑term health impacts, including increases in respiratory conditions, emergency department presentations and mental health service use [28]. Consistent with recent scoping reviews of health system adaptation in Australia, there is growing recognition that workforce preparedness and adaptability are core components of responding to extreme weather challenges [29].

Despite the strong direct associations between bushfire exposure and FTE rates, internal migration did not emerge as a significant mediator. Although a marginally significant indirect effect was observed for severe bushfire exposure, the findings overall suggest that internal migration played only a limited mediating role in the relationship between bushfire exposure and FTE rates. This aligns with broader evidence that long‑term population redistribution following natural disasters is complex and often limited, particularly when communities have strong socio‐economic or cultural ties to place [30]. In the Australian context, there is limited evidence that disaster‐driven migration has resulted in sustained workforce relocation away from affected regions, reinforcing the idea that workforce changes may be more reactive to service demand than to population shifts. This pattern differs from observations in low‐ and middle‐income countries, where disasters frequently drive population displacement and affect health‐workforce allocation [31, 32]. The consistency of these findings across multiple exposure measures increases confidence that the observed relationships are robust to different conceptualisations of bushfire exposure. This reinforces the conclusion that, in Australia, bushfire impacts on workforce supply are driven by health system responses and clinical demand pressures rather than population redistribution.

From a policy perspective, these results suggest that workforce planning should prioritise strengthening health system surge capacity and resilience, particularly in regional and rural areas [33] that are most vulnerable to climate‑driven extremes. Given the limited and inconsistent evidence for an indirect association through internal migration, the findings provide stronger support for considering health‐workforce capacity and service provision when interpreting changes in medical workforce supply following bushfire exposure in our study region. The potential contribution of population movement should nevertheless be considered, particularly where changes in workforce distribution coincide with broader patterns of internal migration. Disaster preparedness frameworks need to incorporate targeted strategies to maintain and rapidly scale clinical workforce capacity, including cross‑jurisdictional support and flexible deployment mechanisms. Given projections of increasing climate hazard intensities (‘cascading and compounding’ impacts) outlined in national climate risk assessments, investment in workforce resilience remains critical for safeguarding health services [34].

### 4.1 Limitations

Several limitations of this study should be acknowledged. First, the use of observational panel data limits causal inference. Unmeasured confounders, such as the speed of infrastructure recovery, disaster relief funding or local workforce initiatives, could partially account for the observed direct association between bushfire exposure and practitioner supply.

Internal migration was measured using annual net migration, which may not fully capture the complexity of population mobility following bushfire exposure. Migration is influenced by a range of economic, social, political, demographic and environmental factors, which often interact with each other [35]. In addition, net migration reflects the balance between population inflows and outflows and does not capture gross population flows, temporary mobility or population turnover. Consequently, the measure used in this study represents only one dimension of population mobility and may not fully capture migration processes relevant to changes in medical workforce distribution.

The relationships between bushfire exposure, population mobility and medical workforce distribution may also develop over different time scales. Previous research highlighted the importance of temporal and historical processes in shaping migration [36]. Although our cumulative bushfire exposure analysis captured the potential influence of repeated exposure over the study period, the annual structure of the data and the mediation framework may not fully capture delayed or dynamic changes in migration and workforce distribution. Future studies with more detailed longitudinal mobility data could examine these temporal relationships more explicitly.

There are also measurement limitations. Socio‐economic status was approximated using SEIFA indices (IRSAD and IER), which, while validated by the ABS [22], capture relative rather than absolute conditions and may not fully reflect the multidimensional nature of socio‐economic disadvantage. Bushfire exposure was based on official disaster records and measured using annual and cumulative event counts. These measures may not fully capture differences in fire intensity, duration, spatial extent, smoke exposure or infrastructure damage. Smaller bushfire events that were not officially recorded may also have been missed, potentially resulting in exposure misclassification. As medical workforce availability was measured as FTE per 100,000 population, changes in the population denominator following bushfire‐related population movement may also influence the observed FTE rate, even when absolute medical practitioner FTE remains unchanged. Furthermore, all data were aggregated at the LGA level, which may mask intra‐regional variation and overlook individual‐level mobility or employment patterns. Differences in geographic size and population density across LGAs, especially in remote areas, could also bias comparisons. Analyses at finer spatial scales or individual‐level data could improve precision and interpretability. It is important to note that although medical practitioner FTE rates provide a useful proxy for system capacity [37], they do not capture service coverage, accessibility or quality critical dimensions of health equity in rural and remote contexts. Future studies should integrate measures of care quality, accessibility and community participation to better understand how bushfire events affect both workforce availability and the functional resilience of local health systems.

## 5 Conclusion

This study investigated the relationship between bushfire exposure and medical practitioner workforce supply across Australian LGAs and assessed whether internal migration mediated this relationship. Across binary, cumulative and severity‐weighted exposure measures, bushfire‐affected areas consistently exhibited higher medical practitioner FTE rates after adjustment for remoteness and socio‐economic factors. Although internal migration was positively associated with practitioner supply, there was limited evidence that it mediated the association between bushfire exposure and workforce distribution. Indirect effects remained close to zero across most models, whereas the severity weighted analysis provided some evidence of a potential indirect effect. These findings suggest that changes in workforce supply following bushfires are primarily driven by direct health system responses to increased service demand rather than by population movement. Strengthening climate‐resilient health‐workforce planning will, therefore, require responsive practitioner deployment, effective retention strategies and targeted support for regional and remote communities that are particularly vulnerable to future climate‐related disasters.

## Author Contributions

**Xin Lin**: software, formal analysis, writing – original draft, visualization. **Ross Bailie**: conceptualisation, methodology, validation, funding acquisition, writing – review and editing. **Peter Radchenko**: conceptualisation, methodology, validation, funding acquisition, writing – review and editing. **Geoffrey Morgan**: conceptualisation, methodology, validation, funding acquisition, writing – review and editing, data curation. **Anirban Chakraborti**: validation, methodology, software, visualisation, writing – review and editing. **K. Shuvo Bakar**: supervision, data curation, software, conceptualisation, methodology, validation, funding acquisition, visualisation, writing – review and editing, project administration.

## Funding

This research received funding from the Sydney Environment Institute (SEI), The University of Sydney, Australia.

## Ethics Statement

The authors have nothing to report.

## Conflicts of Interest

The authors declare no conflicts of interest.

## Data Availability

The data used in this study are publicly available from the relevant Australian government and publicly accessible data sources. The data that support the findings of this study are available at https://www.abs.gov.au/about/data‐services/data‐explorer, https://www.disasterassist.gov.au/find‐a‐disaster/australian‐disasters, https://hwd.health.gov.au/datatool/.
